# Is the combination of bilateral pulmonary nodules and mosaic attenuation on chest CT specific for DIPNECH?

**DOI:** 10.1186/s13023-021-02103-w

**Published:** 2021-11-22

**Authors:** Bilal F. Samhouri, Chi Wan Koo, Eunhee S. Yi, Jay H. Ryu

**Affiliations:** 1 Division of Pulmonary and Critical Care Medicine, Mayo Clinic, Gonda 18 South, Mayo Clinic, 200 First St. SW, Rochester, MN 55905 USA; 2 Department of Radiology, Mayo Clinic, Rochester, MN USA; 3 Division of Anatomic Pathology, Mayo Clinic, Rochester, MN USA

**Keywords:** DIPNECH, Pulmonary nodules, Mosaic attenuation

## Abstract

**Background:**

Diffuse idiopathic pulmonary neuroendocrine cell hyperplasia (DIPNECH) is characterized by multifocal proliferation of pulmonary neuroendocrine cells. On chest CT, DIPNECH exhibits bilateral pulmonary nodules and mosaic attenuation in most patients. We sought to: (1) assess the specificity of this pattern (i.e., bilateral pulmonary nodules together with mosaic attenuation) for DIPNECH; (2) describe its differential diagnosis; and (3) identify the clinico-radiologic features that may help prioritize DIPNECH over other diagnostic considerations.

**Methods:**

We searched the Mayo Clinic records from 2015 to 2019 for patients with bilateral pulmonary nodules and mosaic attenuation on CT who had a diagnostic lung biopsy. A thoracic radiologist reviewed all CT scans. Chi-square test was used for categorical variables, and odds ratios were utilized to measure the association between certain variables and DIPNECH.

**Results:**

Fifty-one patients met our inclusion criteria; 40 (78%) were females and 34 (67%) were never-smokers. Median age was 65 (interquartile range 55–73) years. Lung biopsy was surgical in 21 patients (41%), transbronchial in 17 (33%), and transthoracic in 12 (24%); explanted lungs were examined in 1 (2%). Metastatic/multifocal cancer was the most common diagnosis, and was found in 17 (33%) cases. Bronchiolitis was diagnosed in 12 patients (24%), interstitial lung disease in 10 (20%), and DIPNECH in 5 (10%). Previous diagnosis of an obstructive lung disease (odds ratio 15.8; *P* = 0.002), and peribronchial nodular distribution on CT (odds ratio 14.4; *P* = 0.006) were significantly correlated with DIPNECH. Although statistical significance was not reached, DIPNECH nodules were more likely to display solid attenuations (80% vs. 67%, *P* = 0.45), and were more numerous; > 10 nodules were seen in 80% of DIPNECH cases vs. 52% in others (*P* = 0.23). Because DIPNECH primarily affects women, we analyzed the women-only cohort and found similar results.

**Conclusions:**

Various disorders can manifest the CT pattern of bilateral pulmonary nodules together with mosaic attenuation, and this combination is nonspecific for DIPNECH, which was found in only 10% of our cohort. Previous diagnosis of an obstructive lung disease, and peribronchial distribution of the nodules on CT increased the likelihood of DIPNECH vs. other diagnoses.

**Supplementary Information:**

The online version contains supplementary material available at 10.1186/s13023-021-02103-w.

## Introduction

Pulmonary neuroendocrine cells (PNECs) constitute < 1% of the cells comprising adult human lungs [1]; they are scattered throughout both lungs, and can be seated in the bronchi, or in small airways (e.g., terminal bronchioles and alveolar ducts) [2]. Diffuse idiopathic pulmonary neuroendocrine cell hyperplasia (DIPNECH) is a rare entity that is characterized by abnormal, diffuse, and excessive proliferation of PNECs, and is considered by the World Health Organization as a precursor for other pulmonary neuroendocrine tumors (e.g., carcinoid tumors) [3]. DIPNECH has a strong predilection to affect middle-aged and elderly women, most of whom are never-smokers [4].

On computed tomography (CT) of the chest, DIPNECH exhibits bilateral pulmonary nodules in almost all affected individuals, and mosaic attenuation (“patchwork regions of differing attenuation”) [5] in the majority [4]. Foci of hyperplastic PNECs, with or without carcinoid tumorlets/tumors, are responsible for the diffuse nodules, while constrictive bronchiolitis, which commonly accompanies DIPNECH, is responsible for air-trapping that manifests as mosaic attenuation on CT [4, 6, 7]. This CT pattern is considered the radiologic hallmark of DIPNECH [4, 8].

When DIPNECH is suspected, it can be challenging to confirm the diagnosis; transbronchial and transthoracic needle biopsies are often nondiagnostic, and securing the diagnosis frequently requires a surgical lung biopsy [4, 6], which is an invasive procedure that can be associated with significant morbidity and mortality [9]. This invites the obvious question: can DIPNECH be diagnosed noninvasively (i.e., without biopsy)? In order to answer this key question, we conducted this study with the following objectives: (1) measure the specificity of the CT pattern in-study (i.e., bilateral pulmonary nodules together with mosaic attenuation) for DIPNECH; (2) compare the clinico-radiologic features of patients with DIPNECH against those of patients with disorders other than DIPNECH; and accordingly, (3) formulate an algorithmic approach that aims to aid clinicians in prioritizing the diagnostic possibilities when encountered with the CT pattern in-study.

## Methods

We searched the Mayo Clinic electronic medical records for the terms “mosaic attenuation”, “nodules” and “lung biopsy”. Patients must have had all of the following to meet our inclusion criteria: (1) having undergone a CT exam between 2015 and 2019; (2) demonstrating bilateral pulmonary nodules (excluding calcified nodules thought to represent granulomas) and mosaic attenuation on CT images; and (3) a diagnosis supported by lung biopsy.

To determine the eligibility of patients whose records were retrieved by our search terms, we first reviewed available CT and pathology reports. Patients were deemed eligible if they met all three inclusion criteria outlined above. Chest CT scans of eligible patients were subsequently reviewed by a fellowship-trained thoracic radiologist (C.W.K) with over 10 years of thoracic radiology experience, who re-interpreted all scans and recorded the following: the nodules’ number, density, and lobar and peribronchial distribution; the presence/absence of lung masses; the size of the largest nodule/mass seen; and the presence/absence of bronchial wall thickening. We did not attempt to distinguish between mosaic attenuation secondary to small airways disease vs. pulmonary vascular disease because making such distinction with certainty, though theoretically possible, can be rather challenging [10, 11].

Demographic, clinical, laboratory, pulmonary function and histopathological data were manually extracted. Chest CT scans and pulmonary function tests (PFTs) closest to the date of lung biopsy were selected. Because DIPNECH should be primarily suspected in women [4, 6, 7, 12], we also examined and characterized the women-only cohort.

On PFT, obstructive pattern was defined as forced expiratory volume in 1st second (FEV1)/forced vital capacity (FVC) < lower limit of normal (LLN); restrictive pattern was defined as total lung capacity (TLC) < LLN; mixed obstructive-restrictive pattern was defined as TLC < LLN and FEV1/FVC < LLN [13]; and nonspecific pattern was defined as FEV1 and/or FVC < LLN, with a normal FEV1/FVC ratio, and a TLC value that is ≥ LLN or unavailable [14]. Diffusing capacity of the lungs for carbon monoxide (DLCO) was considered abnormal if DLCO corrected for hemoglobin was < LLN. Air-trapping was defined as residual volume (RV) > 120% predicted, and hyperinflation as TLC > 120% predicted. A positive bronchodilator response was defined as an increase of ≥ 12% and ≥ 200 ml in FEV1 and/or FVC [13].

Frequencies and percentages were used for descriptive statistics. Median and interquartile range (IQR) were used as measures of central tendency. Chi-square test was used for categorical variables. Odds ratios (OR) with 95% confidence intervals (CI) were used to measure the association between different variables and a histopathological diagnosis of DIPNECH. Statistical significance was defined as *P*-value < 0.05. This study was approved by the Mayo Clinic Institutional Review Board.

## Results

Our search terms yielded 141 patients. Ninety patients did not meet our inclusion criteria and were excluded; 28 (31%) had no lung biopsy or a nondiagnostic lung biopsy, whereas 62 (69%) failed to meet our CT inclusion criteria (e.g., unilateral nodules, one nodule only, mosaic attenuation and nodules were not simultaneously present). Fifty-one patients were included in the final analysis; 40 (78%) were women and 11 (22%) were men, with a median age of 64 [IQR 56–73] years (Table 1). Most patients were white (94%), and never-smokers (67%).

**Table 1:** Patient characteristics of the entire cohort (N = 51) and the women-only cohort (N = 40)

	Entire cohort N = 51	Women-only N = 40
Female sex	40 (78)	40 (100)
Age at diagnosis (years); median (IQR)	65 (55–73)	64 (56–72)
Ethnicity		
White	48 (94)	39 (98)
Other	3 (6)	1 (2)
Smoking status		
Never	34 (67)	26 (65)
Previous	14 (27)	12 (30)
Current	3 (6)	2 (4)
Past medical history		
Asthma and/or COPD	7 (14)	7 (18)
Pulmonary hypertension	2 (4)	2 (5)
Previous diagnosis of cancer	16 (31)	8 (20)
Previous diagnosis of autoimmune disease	6 (12)	6 (15)
Lung transplant recipient	4 (8)	2 (5)
Lung biopsy method		
Surgical	21 (42)	14 (35)
Bronchoscopic	17 (33)	14 (35)
Transthoracic (i.e., CT-guided)	12 (24)	11 (28)
Explanted lungs	1 (2)	1 (3)

Data are presented as N (%) unless otherwise specified

*IQR* interquartile range, *COPD* chronic obstructive pulmonary disease, *CT* computed tomography

Past medical history included a previous diagnosis of cancer in 16 patients (31%), autoimmune disease in 6 (12%), and pulmonary hypertension (PH) in 2 (4%); 4 patients were lung transplant recipients. Also, 7 patients (14%) had been previously diagnosed with an obstructive lung disease; asthma in 5, and chronic obstructive pulmonary disease (COPD) in 2.

Twenty-six patients (51%) presented for evaluation of chronic respiratory symptoms namely, dyspnea, cough, or both; median duration of symptoms prior to lung biopsy was 16 [IQR 4–72] months (Table 2). On the other hand, 4 patients (8%) were lung transplant recipients undergoing regular post-transplant surveillance, and 21 (41%) were evaluated for abnormalities noted on radiologic studies performed for other indications. PFT results were available for 43 patients; 4 were lung transplant recipients. Because normal values are based on the recipient’s, rather than the donor’s characteristics (age, height, gender and ethnicity) [13], we only analyzed PFT data belonging to the 39 patients without history of lung transplantation; PFT was abnormal in 69% of cases, and the degree of respiratory impairment was mild to moderate in the majority (71%). Notably, air-trapping was present in only 10 (32%) of 31 patients with RV measurements.

**Table 2:** Presenting symptoms and pulmonary function data across the entire cohort (N = 51) and the women-only cohort (N = 40)

	Entire cohort (N = 51)	Women-only (N = 40)
**Presenting symptom(s)**		
Cough	6 (12)	5 (13)
Cough and dyspnea	7 (14)	6 (15)
Wheezing	1 (2)	1 (3)
Hemoptysis	1 (2)	1 (3)
Dyspnea	11 (22)	10 (25)
Post-transplant surveillance	4 (8)	2 (5)
Incidental CT findings	21 (41)	15 (38)
**Duration of respiratory symptoms, months**		
Median [IQR]	16 [4–72]	19 [4–72]
**Pulmonary function testing**^**¥**^		
PFT pattern	N = 39	N = 33
Normal	12 (31)	11 (33)
Obstructive	10 (26)	9 (27)
Restrictive	11 (28)	7 (21)
Mixed	0 (0)	0 (0)
Nonspecific	6 (15)	6 (18)
Severity of respiratory impairment^¶^	N = 27	N = 22
≥ 70% predicted	11 (41)	8 (36)
60–69% predicted	8 (30)	7 (32)
50–59% predicted	3 (11)	3 (14)
35–49% predicted	3 (11)	2 (9)
< 35% predicted	2 (7)	2 (9)
Air trapping and hyperinflation		
Patients with lung volume measurements	N = 31	N = 26
Air trapping present	10 (32)	9 (35)
Hyperinflation present	3 (10)	3 (12)
Diffusing capacity		
Patients with lung volume measurements	N = 31	N = 26
DLCO reduced	15 (48)	12 (46)
Degree of DLCO reduction (% predicted); median [IQR]	51 [48–61]	50 [45–61]
Bronchodilator responsiveness		
Patients with BD responsiveness testing	N = 26	N = 22
Positive BD response	5 (19)	5 (23)
Negative BD response	21 (81)	17 (77)

Data are presented as N (%) unless otherwise specified

*CT* computed tomography, *IQR* interquartile range, *PFT* pulmonary function test, *FEV1* forced expiratory volume in 1st second, *DLCO* diffusing capacity of carbon monoxide, *BD* bronchodilator

^¥^Excluding the 4 lung transplant recipients

^¶^Severity is determined by FEV1% of predicted value

Lung biopsy was surgical in 21 patients (41%), transbronchial in 17 (33%), and transthoracic (CT-guided) in 12 (24%); histopathological examination was performed on explanted lungs in 1 patient (2%). Histopathology-based diagnoses yielded six broad categories (Table 3): multifocal/metastatic cancer (33%), bronchiolitis (24%), interstitial lung disease (ILD) (20%), DIPNECH (10%), infection (6%), and “other” (8%). Of note, of 17 cancers, 7 (41%) were of pulmonary origin and 10 (59%) were of an extra-pulmonary origin. Although our aim was to calculate the specificity of the CT pattern in-study for DIPNECH, the small number of DIPNECH cases (N = 5) hindered our ability to do so. As such, we will use the term “prevalence” instead of “specificity” hereafter.

**Table 3:** The diagnoses included under the six broad diagnostic categories, with their frequencies across the entire cohort (N = 51) and the women-only cohort (N = 40)

Diagnostic categories	Entire cohort N = 51	Women-only N = 40
Cancer	17 (33)	12 (30)
Metastatic/multifocal lung adenocarcinoma	6 (12)	6 (15)
Metastatic lung cancer (subtype other than adenocarcinoma)	1 (2)	0 (0)
Metastatic adenocarcinoma of unknown primary	1 (2)	1 (3)
Metastatic cancer with primary other than lung	7 (14)	4 (10)
Hematological malignancy	2 (4)	1 (3)
Bronchiolitis	12 (24)	10 (25)
Bronchiolitis obliterans in lung transplant recipient	4 (8)	2 (5)
Bronchiolitis obliterans in non-lung transplant recipient	3 (6)	3 (8)
Follicular bronchiolitis	4 (8)	4 (10)
Respiratory bronchiolitis	1 (2)	1 (3)
Interstitial lung disease	10 (20)	8 (20)
Hypersensitivity pneumonitis	7 (14)	7 (18)
Cicatricial COP	1 (2)	0 (0)
PLCH	1 (2)	1 (3)
UIP	1 (2)	0 (0)
DIPNECH	5 (10)	5 (13)
Infection	3 (6)	2 (5)
Fungal infection	2 (4)	1 (3)
Nontuberculous mycobacteria	1 (2)	1 (3)
Other	4 (8)	3 (8)
Rheumatoid nodules	1 (2)	1 (3)
Sarcoidosis nodules	3 (6)	2 (5)

Data are presented as N (%)

*COP* cryptogenic organizing pneumonia, *PLCH* pulmonary Langerhans cell histiocytosis, *UIP* usual interstitial pneumonia, *DIPNECH* diffuse idiopathic pulmonary neuroendocrine cell hyperplasia

On CT, DIPNECH exhibited a higher number of nodules than other diagnoses, with > 10 nodules in 80% of patients with DIPNECH compared to 52% in others (*P* = 0.23) (Table 4). Also, DIPNECH nodules were more likely to exhibit a peribronchial distribution than others (80% vs. 22%, *P* = 0.006; OR 14.4 [CI 1.4–144]). Among patients whose nodules exhibited a peribronchial distribution, the prevalence of DIPNECH was 29% (*P* = 0.07). Furthermore, DIPNECH nodules were more likely to exhibit solid attenuations (80% vs. 67%, *P* = 0.45). Bronchial wall thickening was seen in all patients with DIPNECH, but only 67% of patients with other diagnoses (*P* = 0.13). In our cohort, 5 patients (10%) had ≥ 1 lung mass (i.e., lesions > 3 cm in diameter) [5], none of whom had DIPNECH. The CT features of each of the six diagnostic categories are depicted in Additional file 1: Table S1.

**Table 4:** Chest CT scan findings in patients with DIPNECH vs. patients with other diagnoses

Characteristic	DIPNECH N = 5	Other diagnoses N = 46
Number of nodules		
2–3	0 (0)	7 (15)
4–5	0 (0)	3 (7)
6–10	1 (20)	12 (26)
> 10	4 (80)	24 (52)
Nodule density		
Solid only	4 (80)	29 (63)
Solid and subsolid^**†**^	1 (20)	17 (37)
Lobar predominance		
Upper lobes	0 (0)	3 (7)
Lower lobes	0 (0)	2 (4)
Random	5 (100)	41 (89)
Peribronchial distribution**	4 (80)	10 (22)
Bronchial wall thickening	5 (100)	31 (67)
Masses present	0 (0)	5 (11)
Diameter of largest nodule/mass (mm); median (range)	7 (4–30)	8 (3–69)

Data are presented as N (%) unless otherwise specified

DIPNECH: diffuse idiopathic pulmonary neuroendocrine cell hyperplasia

^†^Subsolid attenuations include ground-glass, and part-solid attenuations

^**^Signifies *p*-value < 0.05

When the data analysis was limited to the women-only cohort (N = 40), median age was 64 [IQR 56–72] years, most were white (98%), and 65% were never-smokers (Table 1). Past medical history included a previous diagnosis of cancer in 8 patients (20%), autoimmune disease in 6 (15%), an obstructive lung disease in 7 (18%), and PH in 2 (5%); 2 were lung transplant recipients. Among women, multifocal/metastatic cancer remained the most common diagnostic category (30%), followed by bronchiolitis (25%), ILD (20%), DIPNECH (13%), infection (5%), and “other” (8%) (Table 3). Six (50%) of the 12 cancers diagnosed in women were of pulmonary origin, all of which were adenocarcinomas.

Several observations underscore the value of the clinical context including the past medical history in the diagnostic evaluation of patients exhibiting the CT pattern in-study. In our cohort, a previous diagnosis of an obstructive lung disease (i.e., asthma or COPD) was more commonly encountered among patients with DIPNECH (60% vs. 9%, *P* = 0.002), and the presence of such history significantly correlated with an eventual diagnosis of DIPNECH on histopathology (OR 15.8 [CI 2.0–124]). Importantly, this correlation retained statistical significance in the women-only cohort as well (*P* = 0.008).

Of 12 women in whom lung biopsy showed multifocal/metastatic cancer, 8 (67%) had a known history of cancer when the CT abnormalities were identified; in the 4 remaining women, metastatic/multifocal lung adenocarcinoma was diagnosed in 3, and lymphoma in 1. Further, both women with an infectious etiology were known to be taking immunosuppressive medications, and both women with history of lung transplantation were ultimately diagnosed with bronchiolitis obliterans syndrome.

Four women were diagnosed with follicular bronchiolitis in our cohort. When the CT pattern in-study was identified in these patients, all had been known to have history of autoimmunity; 1 patient had scleroderma, 1 had rheumatoid arthritis (RA), and the other 2 were known to have persistently elevated titers of various autoantibodies (antinuclear, anti-Scl70 and anti-CCP antibodies in one, and anti-RNP antibodies in one). Similarly, the woman with rheumatoid lung nodules had been diagnosed with RA several years preceding the CT abnormalities.

Two patients in our cohort had PH which, similar to air-trapping, can manifest mosaic attenuation on chest CT [10]. Both patients manifested PFT abnormalities (airflow obstruction in one, and nonspecific pattern with air-trapping in the other), and were ultimately diagnosed with sarcoidosis and follicular bronchiolitis, respectively. We believe that, in both patients, the mosaic attenuation observed is at least partly related to air-trapping and may not be solely attributed to PH.

Lastly, the patient with a usual interstitial pneumonia (UIP) pattern on histopathology had a CT pattern that is “indeterminate for UIP” owing to the degree of bronchocentric fibrosis. This patient was also found to have elevated autoantibody titers; in particular, antinuclear, anti-U1 RNP, and anti-NXP-2 antibodies.

## Discussion

This is the first study to examine the prevalence of DIPNECH among patients exhibiting the CT pattern that is considered the radiologic hallmark of DIPNECH, i.e., bilateral pulmonary nodules combined with mosaic attenuation. We found that the prevalence of DIPNECH was only 10% in our cohort, and that this CT pattern can be encountered in the context of a broad array of disorders including: metastatic/multifocal cancer, bronchiolitis, ILD, atypical infections, and autoimmune/connective tissue diseases (CTDs). Interestingly, individuals who had a previous diagnosis of an obstructive lung disease, and those in whom the pulmonary nodules exhibited a peribronchial distribution were more likely to have DIPNECH.

Our cohort predominantly comprised middle-aged and elderly women, most of whom were never-smokers; many presented with chronic respiratory symptoms, namely dyspnea, cough, or both. These patient characteristics are reminiscent of those typically affected by DIPNECH [4, 6, 7, 12]. Accordingly, the present study is well-positioned to elucidate the alternative diagnoses that should be considered in patients presenting with clinical and radiologic findings suggestive of DIPNECH.

**Fig. 1:**
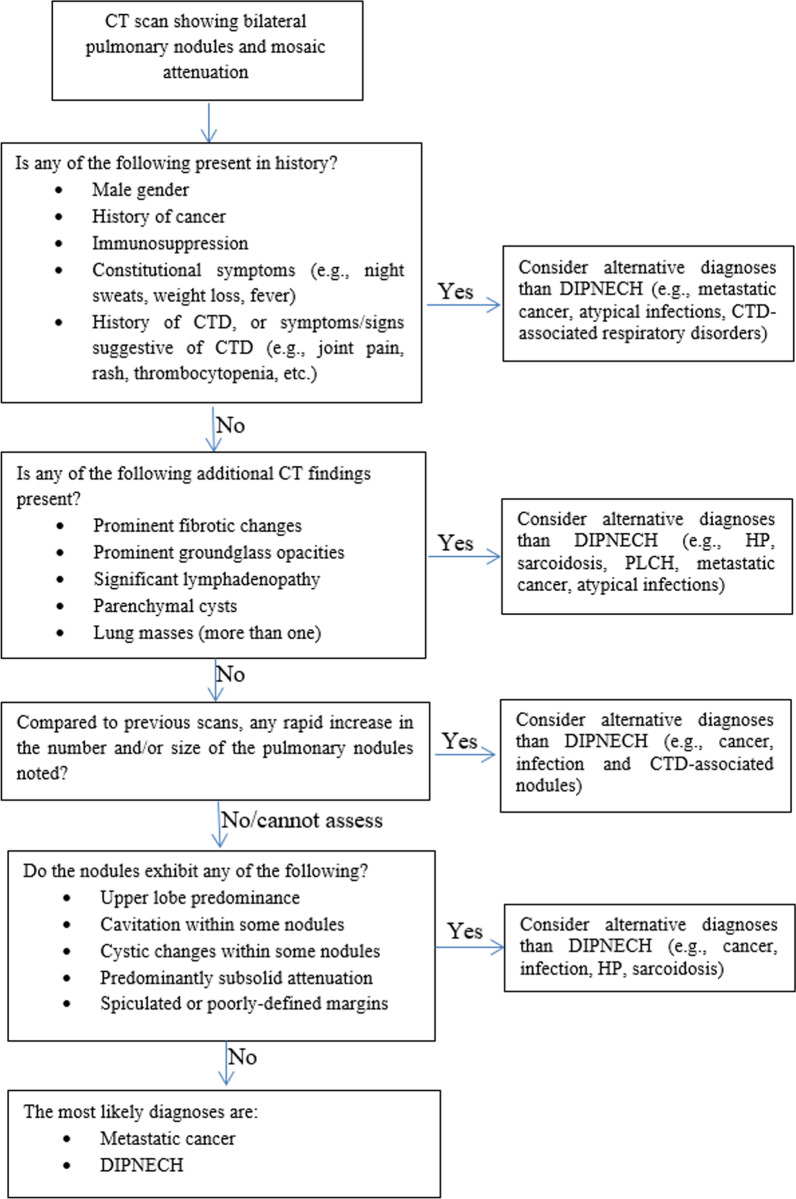
Algorithmic approach towards CT scans depicting bilateral pulmonary nodules and mosaic attenuation. Abbreviations: CT: Computed tomography, CTD: connective tissue disease, DIPNECH: diffuse idiopathic pulmonary neuroendocrine cell hyperplasia, HP: hypersensitivity pneumonitis, PLCH: pulmonary Langerhans cell histiocytosis

Our data suggest that the CT pattern under-study is nonspecific for DIPNECH and although meticulous examination of the CT images may narrow down the diagnostic possibilities, it cannot be diagnostic without carefully considering the clinical context and the radiologic evolution of the nodules over time. While a comprehensive discussion relating to the clinical and radiologic features of each differential diagnosis is beyond the scope of this paper, we will highlight certain high-yield features and propose an algorithmic approach (Fig. 1) that integrates clinical and radiologic data and might prove helpful in distinguishing DIPNECH from other diagnoses.

The importance of accurately assessing the clinical context through careful history-taking cannot be overstated. Limited awareness of DIPNECH as an entity among clinicians and radiologists, complicated by the nonspecific symptomatology of DIPNECH have led to substantial delays (a decade or more in 50–60% of patients) [4, 6] and errors in its diagnosis; nearly one-half of patients in previous studies [4, 6, 7], and 60% in the present study had had their respiratory syndromes erroneously attributed to asthma and/or COPD before a final diagnosis of DIPNECH was attained. In the present study, patients with DIPNECH were significantly more likely to have been misdiagnosed as asthma or COPD. In other words, in patients with bilateral pulmonary nodules and mosaic attenuation on CT, carrying a diagnosis of asthma or COPD renders an eventual diagnosis of DIPNECH more likely.

In patients with primary lung cancer, the interval between symptom onset and diagnosis is noticeably shorter than in DIPNECH, and is on the order of weeks to months [15]. In patients with pulmonary metastases of an extrapulmonary origin, however, respiratory symptoms are usually absent, and systemic symptoms dominate instead [16].

DIPNECH is seldom associated with systemic symptoms (e.g., loss of appetite, weight loss, fever, night sweats), and aside from metastatic cancers, this also contrasts disseminated fungal infections wherein such symptoms are frequently encountered [16, 17]. In contrast to CTDs, atypical infections, and metastatic cancers, all of which commonly involve other organ systems, DIPNECH, per se, is strictly limited to the lungs. An exception to this rule, however, is when DIPNECH is complicated by metastatic carcinoid tumor [4, 6]. While DIPNECH is typically encountered in nonsmokers, some of the other diagnostic considerations included herein affect smokers almost exclusively (e.g., pulmonary Langerhans cell histiocytosis (PLCH) and respiratory bronchiolitis) [18, 19].

In our women-only cohort, all patients with CTD-associated respiratory manifestations, particularly follicular bronchiolitis and rheumatoid nodules, had been known to have underlying autoimmune disease prior to their diagnostic evaluation. This is concordant with previous studies which showed that CTD-associated respiratory manifestations tend to appear later in the course of a known CTD, rather than as the initial presenting features of a previously-undiagnosed CTD [20–22].

Having a history of cancer in an individual manifesting multiple pulmonary nodules on CT should prompt consideration that these nodules are cancerous until proven otherwise. This proposition is supported by our study; most women who were diagnosed to have metastatic/multifocal cancer had a previous diagnosis of cancer. Furthermore, in one study that included 228 patients with one or more pulmonary nodule(s) and a previous diagnosis of an extrapulmonary malignancy, cancer was found in 90% of lung biopsies; 64% were metastatic, and 26% were primary lung cancers [23]. In the same study, compared to a single nodule, the presence of multiple nodules further increased the likelihood that those nodules represented metastases. It is prudent to keep in mind that although a history of cancer is present in most patients with pulmonary metastases, such history may be lacking in some cases, and pulmonary metastases can be encountered as the initial manifestation of a previously-undiagnosed malignancy [24].

**Fig. 2:**
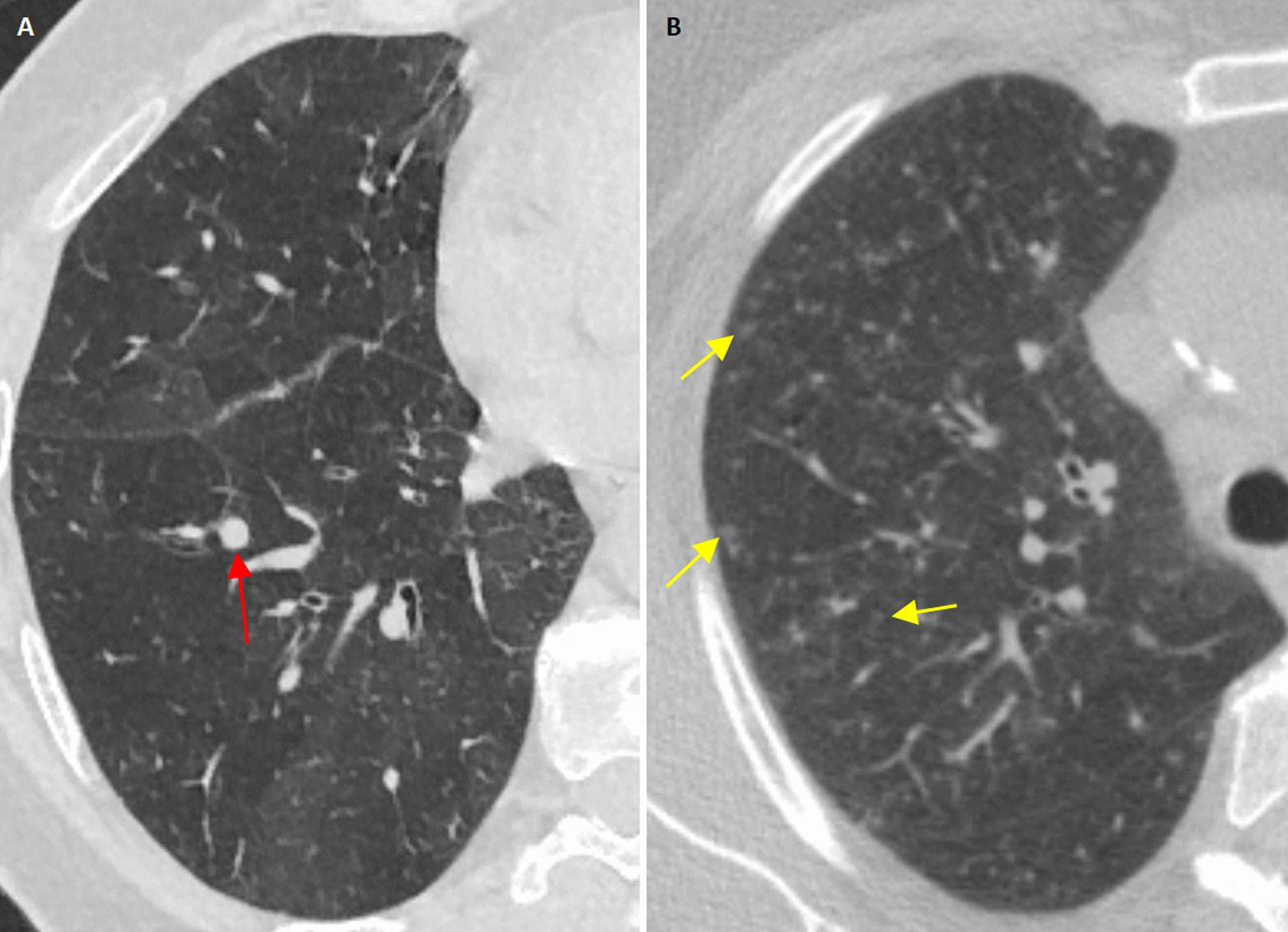
Illustrative chest CT scans. **A** Depicts a chest CT scan obtained from a 69 year-old female with DIPNECH. Note the solid and round nodule with smooth and well-defined margins (red arrow), in addition to pronounced mosaic attenuation. **B** Depicts a chest CT scan obtained from a 69 year-old female with respiratory bronchiolitis. Note the centrilobular, subsolid, hazy and ill-defined micronodules (yellow arrows), in addition to mosaic attenuation

In terms of the characteristics of the nodules themselves, the present study found that DIPNECH nodules exhibit a solid attenuation in most cases. According to Carr et al., DIPNECH nodules are well-defined, round or oval in shape, rarely calcified, and noncavitary (Fig. 2a) [6]. These features are grossly disparate from HP and respiratory bronchiolitis whose nodules are subsolid, hazy and ill-defined (Fig. 2b) [25, 26]. The recognition of spiculated margins, and/or cystic or cavitary components within the nodules favors a diagnosis of cancer [27–30]. In addition to malignancies, cavitary nodules can be seen in association with fungal infections and RA [22, 31].

In DIPNECH, the majority of nodules are 6–10 mm in diameter [6]. This differs from HP, bronchiolitis and sarcoidosis, wherein micronodules (i.e., nodular opacities ≤ 3–7 mm in diameter) [5] are dominant [25, 26]. Notably, lung masses are rarely encountered in DIPNECH [4], and when present, they likely represent carcinoid tumors. In a study that included 32 patients with DIPNECH, only 1 lung mass was found [7]. While identifying one lung mass does not rule out DIPNECH, identifying multiple masses should invite serious consideration of an alternative diagnosis. In the present study, 5 patients had at least one lung mass, none of whom had DIPNECH.

Based on the present study, a peribronchial distribution of the nodules appears to be the CT feature most predictive of DIPNECH, when occurring in combination with mosaic attenuation. In the present study, we found that DIPNECH nodules exhibit a random craniocaudal distribution. Conversely, two larger studies reported that DIPNECH nodules display an affinity to involve lower- and mid-lung zones [6, 7]. Nonetheless, such distribution differs from HP and sarcoidosis where upper- and mid-lung zones are preferentially involved [25, 32].

On CT, metastatic cancers with hematogenous seeding of the lungs classically appear as nodular lesion(s) that can be single to innumerable, and can range from few millimeters to several centimeters in size. Typically, these nodules are of solid attenuation, round in shape with smooth and well-defined borders, and are mostly noncalcified and noncavitary. Also, they favor peripheral and lower lung zones [28, 29]. These radiologic features are similar, to a large extent, to DIPNECH [6]; consequently, making the distinction between DIPNECH and pulmonary metastases solely on the basis of chest CT may not be possible.

Like DIPNECH, multifocal adenocarcinoma (MAC) of the lung primarily affects nonsmoker women, and manifests multiple, slowly-progressive pulmonary nodules that can be centered around the airways, with a predilection to involve peripheral lung zones [33]. However, two key features help distinguish MAC from DIPNECH; in MAC, the nodules are considerably less in number, and more likely to exhibit subsolid attenuations. In the study by Nakata et al., 31 patients with MAC had a total of 68 nodules on CT, only 19 (28%) of which were solid [34]. In another study, 39 patients with MAC had 149 nodules on CT, averaging 3.8 nodules per patient [35].

Multiple parenchymal cysts, extensive pulmonary fibrosis, diffuse ground-glass opacities, pleural effusions, and prominent intra- or extra-thoracic lymphadenopathy are very unusual findings in DIPNECH [4, 6, 7], and their presence urges considering alternative diagnoses, such as PLCH (parenchymal cysts), HP (fibrosis and ground-glass opacities), sarcoidosis (fibrosis and lymphadenopathy), infections, and malignancies (lymphadenopathy and pleural effusions).

Whenever previous imaging is available, comparison to assess the temporal evolution of the nodules over time is necessary. In the study by Little et al., 30 patients with DIPNECH had their initial and follow-up chest CT scans reviewed; over a median interval of 3.4 years, no growth was noted in the dominant nodule in one-third of cases, whereas slight growth (mean increase in size was 3.6 mm) was noted in the remainder two-thirds [7]. In view of this rather indolent nature, a rapid increase in the number and/or size of pulmonary nodules renders DIPNECH highly unlikely, and is more consistent with an infectious, inflammatory, or malignant etiology [36].

On CT imaging, mosaic attenuation is most commonly observed in the context of disorders that involve the small airways, such as primary bronchiolitides, in addition to certain ILDs, infections, and CTD-related manifestations (e.g., sarcoidosis nodules) [10]. In our study, however, metastatic/multifocal cancer was the most prevalent diagnosis. This observation is not entirely surprising because: (1) cancer may originate from cells situated within the small airways (e.g., lung adenocarcinoma) [37]; and (2) intra-airway metastasis from pulmonary and extra-pulmonary origins is a well-documented, though rare, phenomenon [30]. Although the mosaic attenuation seen in those patients with cancer may be secondary to another respiratory disorder, such possibility seems unlikely since none of them had been diagnosed with a chronic lung disease.

Our study has limitations. Requiring a lung biopsy for inclusion may have excluded patients with common disorders and those with classic CT appearances in whom the diagnosis could be established noninvasively. The previous notion, compounded by the referral nature of our center may have resulted in under-representation of common disorders, and over-representation of rare disorders, including DIPNECH. As a result, our study may have overestimated the specificity of the pattern in-study for DIPNECH. Finally, the modest sample size may have hindered our ability to achieve statistical significance in some instances.

## Conclusion

The CT pattern, bilateral pulmonary nodules together with mosaic attenuation, is nonspecific for DIPNECH and can be seen in a myriad of disorders. Although the prevalence of DIPNECH in our cohort was low (10%), it may still be an overestimation owing to the referral nature of our center in addition to our inclusion criteria that necessitated lung biopsy. Amongst individuals manifesting the CT pattern in-study, the likelihood of DIPNECH increases if a previous diagnosis of an obstructive lung disease is present, and if the nodules exhibit a peribronchial distribution. Careful assessment of the clinical context, in addition to the morphology, distribution, and temporal evolution of the nodules helps prioritize the diagnostic possibilities. In very select circumstances, such linic-radiologic evaluation may be sufficiently suggestive of DIPNECH, potentially obviating the need for lung biopsy.

## Supplementary Information

**Additional file 1**. Chest CT scan findings divided by diagnostic category across the entire cohort (n = 51).

## Data Availability

The data that support the findings of this study are available from Mayo Clinic but restrictions apply to the availability of these data, which were used under license for the current study, and so are not publicly available. Data are however available from the authors upon reasonable request and with permission of the Mayo Clinic’s IRB.

## Abbreviations

COPD: Chronic obstructive pulmonary disease
CT: Computed tomography
CTD: Connective tissue disease
CI: Confidence interval
DIPNECH: Diffuse idiopathic pulmonary neuroendocrine cell hyperplasia
DLCO: Diffusing capacity of the lungs for carbon monoxide
FEV1: Forced expiratory volume in 1st second
FVC: Forced vital capacity
HP: Hypersensitivity pneumonitis
ILD: Interstitial lung disease
IQR: Interquartile range
IRB: Institutional review board
LLN: Lower limit of normal
MAC: Multifocal adenocarcinoma
OR: Odds ratio
PFT: Pulmonary function test
PH: Pulmonary hypertension
PLCH: Pulmonary Langerhans cell histiocytosis
PNEC: Pulmonary neuroendocrine cell
RA: Rheumatoid arthritis
RV: Residual volume
TLC: Total lung capacity
UIP: Usual interstitial pneumonia
