# Supplementary material for: Is the combination of bilateral pulmonary nodules and mosaic attenuation on chest CT specific for DIPNECH?
Source: Orphanet J Rare Dis. 2021 Nov 22;16:490. doi: 10.1186/s13023-021-02103-w (PMC8607646; doi:10.1186/s13023-021-02103-w)
Supplement: Supplementary file 1 — Additional file 1. Chest CT scan findings divided by diagnostic category across the entire cohort (n = 51). [file 13023_2021_2103_MOESM1_ESM.docx]

**Additional file 1.** Chest CT scan findings divided by diagnostic category across the entire cohort (n=51).

|  | **Cancer**  **N=17** | **Bronchiolitis**  **N=12** | **ILD**  **N=10** | **DIPNECH**  **N=5** | **Infection**  **N=3** | **Other**  **N=4** |
| --- | --- | --- | --- | --- | --- | --- |
| **Number of nodules** | | | | | | |
| 2-3 | 0 (0) | 3 (25) | 2 (20) | 0 (0) | 2 (67) | 0 (0) |
| 4-5 | 1 (6) | 1 (8) | 1 (10) | 0 (0) | 0 (0) | 0 (0) |
| 6-10 | 4 (24) | 3 (25) | 4 (40) | 1 (20) | 0 (0) | 1 (25) |
| >10 | 12 (71) | 5 (42) | 3 (30) | 4 (80) | 1 (33) | 3 (75) |
| **Nodule density** | | | | | | |
| Solid only | 12 (71) | 6 (50) | 5 (50) | 4 (80) | 2 (67) | 4 (100) |
| Solid and subsolid | 5 (29) | 6 (50) | 5 (50) | 1 (20) | 1 (33) | 0 (0) |
| **Lobar predominance** | | | | | | |
| Upper lobes | 0 (0) | 2 (17) | 0 (0) | 0 (0) | 1 (33) | 0 (0) |
| Lower lobes | 2 (12) | 0 (0) | 0 (0) | 0 (0) | 0 (0) | 0 (0) |
| Random | 15 (88) | 10 (83) | 10 (100) | 5 (100) | 2 (67) | 4 (100) |
| **Peribronchial distribution** | 1 (6) | 5 (42) | 3 (30) | 4 (80) | 1 (33) | 0 (0) |
| **Bronchial wall thickening** | 11 (65) | 12 (100) | 5 (50) | 5 (100) | 2 (67) | 1 (25) |
| **≥1 Lung mass present** | 4 (24) | 0 (0) | 0 (0) | 0 (0) | 1 (33) | 0 (0) |
| **Diameter of largest nodule/mass (mm); median (range)** | 21 (5-69) | 7 (3-18) | 7 (4-12) | 7 (4-30) | 6 (4-8) | 10 (6-21) |

Data are presented as N (%) unless otherwise specified.

**Abbreviations.** ILD: interstitial lung disease; DIPNECH: diffuse idiopathic pulmonary neuroendocrine cell hyperplasia.
